# Effluent Tenascin-C Levels Reflect Peritoneal Deterioration in Peritoneal Dialysis: MAJOR IN PD Study

**DOI:** 10.1155/2015/241098

**Published:** 2015-12-06

**Authors:** Ichiro Hirahara, Eiji Kusano, Toshimi Imai, Yoshiyuki Morishita, Makoto Inoue, Tetsu Akimoto, Osamu Saito, Shigeaki Muto, Daisuke Nagata

**Affiliations:** Division of Nephrology, Department of Internal Medicine, Jichi Medical University, 3311-1 Yakushiji, Shimotsuke, Tochigi 329-0498, Japan

## Abstract

Peritoneal deterioration causing structural changes and functional decline is a major complication of peritoneal dialysis (PD). The aim of this study was to explore effluent biomarkers reflecting peritoneal deterioration. In an animal study, rats were intraperitoneally administered with PD fluids adding 20 mM methylglyoxal (MGO) or 20 mM formaldehyde (FA) every day for 21 days. In the MGO-treated rats, tenascin-C (TN-C) levels in the peritoneal effluents were remarkably high and a cluster of TN-C-positive mesothelial cells with epithelial-to-mesenchymal transition- (EMT-) like change excessively proliferated at the peritoneal surface, but not in the FA-treated rats. Effluent matrix metalloproteinase-2 (MMP-2) levels increased in both the MGO- and FA-treated rats. In a clinical study at 18 centers between 2006 and 2013, effluent TN-C and MMP-2 levels were quantified in 182 PD patients with end-stage renal disease. Peritoneal function was estimated using the peritoneal equilibration test (PET). From the PET results, the D/P Cr ratio was correlated with effluent levels of TN-C (*ρ* = 0.57, *p* < 0.001) and MMP-2 (*ρ* = 0.73, *p* < 0.001). We suggest that TN-C in the effluents may be a diagnostic marker for peritoneal deterioration with EMT-like change in mesothelial cells in PD.

## 1. Introduction

Peritoneal dialysis (PD) is a treatment for patients with severely reduced or absent renal function. Long-term PD results in peritoneal deterioration, causing structural changes and functional decline such as increased peritoneal solute transport; this leads to ultrafiltration failure of the peritoneal membrane. Peritoneal deterioration may result in the cessation of PD treatment; this complication leads to encapsulating peritoneal sclerosis (EPS) in patients with long-term history of PD for renal failure. EPS is associated with an extremely high mortality rate [1–4]. Safe and effective PD requires monitoring of peritoneal deterioration developing to EPS.

Functional decline of the peritoneum, such as increasing peritoneal transport rate, can be assessed with the peritoneal equilibration test (PET) [4–6]. The transport rate increases with peritoneal deterioration and a higher transporter membrane state is a factor contributing to the occurrence of EPS in patients who have experienced PD treatment [5]. Structural changes of the peritoneum can be examined by biopsy of peritoneal tissue; however, because it is invasive, this method is inappropriate for continuous testing. Only exceptionally skilled clinical pathologists perform cytodiagnosis of mesothelial cells in peritoneal effluents [4]. Easy and noninvasive methods are required to evaluate peritoneal structural changes for diagnosis of peritoneal deterioration.

Some effluent biomarkers, such as matrix metalloproteinase-2 (MMP-2), interleukin-6 (IL-6), hyaluronan, and cancer antigen-125 (CA125), are measured to estimate peritoneal deterioration or progression to EPS during PD [2, 4, 7–11]. Kaku et al. demonstrated that the correlation coefficient between the peritoneal solute transport rate estimated by the PET and the effluent levels of MMP-2 was higher than those of IL-6, hyaluronate, and CA125 [11]. We also reported that effluent MMP-2 levels were high in the patients with peritoneal deterioration and strongly correlated with the results of the PET [8]. MMP-2 degrades components of the extracellular matrix, such as fibronectin and type IV collagen, which comprise the basement membrane. MMP-2 is produced by mesenchymal cells, macrophages, and endothelial cells in the peritoneum; it plays important roles in angiogenesis, epithelial-to-mesenchymal transition (EMT) of mesothelial cells, and migration of cells that promote inflammation or fibroplasia [2, 7, 9–15]. Because they reflect peritoneal deterioration with structural changes and functional decline, effluent MMP-2 levels may be valuable in predicting the occurrence of EPS. Thus, MMP-2 may be the best available indicator of peritoneal deterioration.

Morphologically, mesothelial cells change from polygonal cobblestone-like appearance to a spindle-shaped form in PD [16]. Aguilera et al. reported that the EMT of mesothelial cells may be involved in triggering peritoneal injury with fibrosis in PD patients [17]. In addition, high solute transport of peritoneal membrane is associated with EMT of mesothelial cells [18]. These reports suggest that monitoring EMT of mesothelial cells may enable early diagnosis of peritoneal deterioration.

Tenascin-C (TN-C) is an extracellular matrix glycoprotein formed by hexamers of approximately 300 kDa subunits. Although expression of TN-C is rare in normal adult tissues, high levels are observed in pathological states featuring tissue remodeling, such as inflammation, wound healing, and cancer progression. In particular, TN-C is expected as a biomarker for myocarditis or aortic aneurism [19]. In addition, TN-C induces EMT-like change in cancer cells [20, 21]. As mentioned above, EMT-like change in mesothelial cells is induced at early stage of peritoneal deterioration. TN-C may have potential as an indicator of tissue injury; however, to the best of our knowledge, there are no reports concerning TN-C in PD.

This study aimed to investigate simple and noninvasive methods, to evaluate indicators of peritoneal deterioration used to diagnose early peritoneal deterioration with structural changes and functional decline, such as high solute transport of the peritoneal membrane. Therefore, we examined whether TN-C and MMP-2 in the peritoneal effluents have potential as indicators of peritoneal deterioration.

## 2. Methods and Patients

### 2.1. Preparation of Animal Models of Peritoneal Injury

Animals used in this study were 5-6-week-old male Sprague-Dawley (SD) rats weighing approximately 200–250 g (Charles River Japan, Kanagawa, Japan). They were housed in cages in an air-conditioned room, which was maintained at a constant temperature of 23 ± 2°C and relative humidity of 50 ± 10%. The animals were kept under a 12-hour light/dark cycle and had free access to sufficient pellet food and water.

The PD fluids were prepared by adding 20 mM methylglyoxal (MGO) or 20 mM formaldehyde (FA) to a solution (2.5% glucose, 100 mM NaCl, 35 mM sodium lactate, 2 mM CaCl_2_, and 0.7 mM MgCl_2_, pH 5.0), which were then daily sterilized by filtration just before injection. Rats were divided into three groups (*n* = 6/group) and intraperitoneally administered with the following solutions for 21 days: group 1, 100 mL/kg PD fluid without adding MGO or FA; group 2, 100 mL/kg PD fluid containing 20 mM MGO; group 3, 100 mL/kg PD fluid containing 20 mM FA. MGO and FA concentrations were determined based on previous reports [12–14]. If any solution remained in the peritoneal cavity, it was drained prior to injection. On day 22 after the start of the experiment, 50 mL/kg of PD fluid containing 2.5% glucose (Midperiq L250, Terumo Co., Tokyo, Japan) was intraperitoneally injected and the drained dialysate was collected 90 minutes later to analyze TN-C and MMP-2 in the effluents. The parietal peritoneum was also sampled for histological analysis.

Adequate attention was paid to maintaining a hygienic environment and to preventing infectious peritonitis in the animals. Furthermore, a sterility test was performed using the dialysate drained on day 22 to check for the presence of aerobic bacteria, anaerobic bacteria, and fungi. All rats were confirmed to be uninfected.

The Institutional Animal Experiment Committee of Jichi Medical University approved the protocol of this animal study. The animal experiments were conducted in accordance with the Institutional Regulations for Animal Experiments and Fundamental Guidelines for the Proper Conduct of Animal Experiments and Related Activities in Academic Research Institutions under the jurisdiction of Japan's Ministry of Education, Culture, Sports, Science and Technology.

### 2.2. Immunohistological Analysis of Peritoneum in Animal Study

The parietal peritoneum was sampled from the corresponding sites of each rat and fixed with 10% FA/0.1 M phosphate buffer (pH 7.2). The peritoneal specimens were embedded in paraffin to prepare tissue sections with a thickness of 2-3 *μ*m. To analyze the thickness of the peritoneum, the sections were sliced perpendicular to the peritoneal surface. The peritoneal tissue sections prepared from a paraffin block were dewaxed with xylene. The sections were treated with 0.4% pepsin/0.01 N HCl for 10 minutes at 37°C, followed by treatment with 0.3% H_2_O_2_/methanol for 30 minutes at room temperature. After blocking with 1% bovine serum albumin for 20 minutes at room temperature, these sections were treated overnight at 7°C with a monoclonal antibody against TN-C (Immuno-Biological Laboratories Co., Ltd., Gunma, Japan) at a dilution of 1 : 500, followed by staining with biotinylated anti-mouse IgG (Dako Cytomation Denmark A/S, Glostrup, Denmark) for 30 minutes at room temperature. The sections were treated with peroxidase-labeled streptavidin (Dako) at a dilution of 1 : 700 for 30 minutes at room temperature and then peroxidase activity was detected with 3,3′-diaminobenzidine tetrahydrochloride (Sigma, St. Louis, MO, USA). Sections were also counterstained with Meyer's hematoxylin. Negative staining was confirmed by incubation without primary antibody for immunohistochemical staining.

### 2.3. Analysis of the Drained Dialysate in Animal Study

To explore markers for peritoneal deterioration, TN-C levels in the drained dialysate were quantitatively determined by enzyme-linked immunosorbent assay (ELISA) (Immuno-Biological Laboratories Co., Ltd.). MMP-2 in the dialysate was analyzed by gelatin zymography [7, 12, 13]. In brief, after electrophoresis under nonreducing conditions on 8% polyacrylamide gels containing 1 mg/mL gelatin, the gels were treated with 2.5% Triton-100/0.1 M NaCl/50 mM Tris-HCl (pH 7.5) for 2 hours and incubated for 18 hours at 37°C in 50 mM Tris-HCl (pH 7.5)/10 mM CaCl_2_. The gels were then stained with 0.1% Coomassie Brilliant Blue. MMP-2 was detected as unstained 64 kDa proteolytic bands in the stained gels. The relative concentrations of MMP-2 in the dialysate were quantified by scanning proteolytic bands on the zymograms using the ImageJ quantitation software program (National Institutes of Health, Bethesda, MD, USA).

### 2.4. Patients in Multicenter Clinical Study

PD patients with end-stage renal disease at 18 centers in Japan were analyzed during the period from January 2007 to March 2013. Patients treated with PD for less than 3 months were excluded from the present study, as were those with bacterial peritonitis at the time of the analysis or in the 4 preceding weeks.

Clinical analysis was conducted after receiving approval from the Ethics Committee of Jichi Medical University, and informed consent was obtained from each patient. TN-C in the serum was analyzed in 20 patients from whom informed consent for analysis of the serum had been obtained.

This study was registered as the MAJOR IN PD study (Multicenter Analysis in Japan, ORiginal INdicator of Peritoneal Deterioration) in the University Hospital Medical Information Network-Clinical Trials Registry (UMIN-CTR), which was approved by the International Committee of Medical Journal Editors (number UMIN000010572).

### 2.5. Peritoneal Equilibration Test (PET) for Human

The peritoneal solute transport rate was assessed by the PET [4, 6]. Intra-abdominal fluid was drained and PD fluid containing 2.27–2.5% glucose was intraperitoneally injected. Creatinine (Cr) level of the drained dialysate obtained 4 hours after injection (D) was divided by that of the patient's blood (P) to calculate the D/P Cr ratio. The glucose level of the dialysate obtained 4 hours after injection (D) was divided by that obtained immediately after injection (D0) to calculate the D/D0 glucose ratio. The drained dialysate was aliquoted and stored at −80°C. It was confirmed that TN-C and MMP-2 were stable at −80°C and were not decomposed by several repeated freeze-thaw cycles.

### 2.6. Analysis of Biomarker Levels in the Drained Dialysate in Multicenter Clinical Study

The concentrations of TN-C and MMP-2 in the drained dialysate obtained at the PET were measured by EIA (TN-C: Immuno-Biological Laboratories Co., Ltd.; MMP-2: GE Healthcare, Piscataway, NJ, USA).

Serum TN-C levels were analyzed and regression lines were calculated based on the power relationship between the molecular weights of *β*2-microglobulin (MW: 11,800 Da), MMP-3 (MW: 59,000 Da), albumin (MW: 69,000 Da), transferrin (MW: 85,000 Da), and IgG (MW: 150,000 Da) and their measured dialysate/serum (D/S) ratios when plotted on a double-logarithmic scale [8–10]. These proteins were transported from the circulation to the peritoneal cavity by the osmotic pressure of the PD fluid. By interpolation of the molecular weights of TN-C (MW: 1,800,000 Da) in the regression equation, the expected D/S ratios were calculated, assuming that their concentration in the drained dialysate would be determined by transport only from the circulation.

### 2.7. Statistical Analysis

Statistical analyses were performed using R statistical software version 2.15.1 (R Foundation for Statistical Computing). Receiver operating characteristic (ROC) curve analyses were performed to evaluate the diagnostic accuracy of MMP-2 and TN-C. Comparisons between groups were performed by Wilcoxon's test. Relationships between clinical variables and effluent biomarker levels were analyzed by Spearman's correlation coefficient test. Clinical data are expressed as medians with the spread from the 25th to 75th percentiles. In the animal experiment, statistical comparisons were conducted by analysis of variance (ANOVA). Animal experimental data are presented as the mean ± SD. A *p* value of less than 0.05 was considered to be statistically significant.

## 3. Results

### 3.1. Animal Experiments

**Figure 1:**
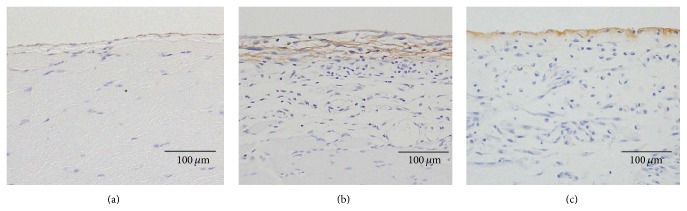
Immunohistopathological findings of peritoneum of the MGO- or FA-treated rats. The parietal peritoneum was analyzed histologically by immune staining with anti-TN-C antibody. (a) Control rat. (b) MGO-treated rat. (c) FA-treated rat. ×200.

In rats receiving PD fluids containing 20 mM MGO or 20 mM FA, peritoneal thickening significantly increased (Figure 1). These results confirmed that both the MGO- and FA-treated rats had induced peritoneal deterioration with structural changes.

In control rats, a monolayer of mesothelial cells was observed on the surface of the thin peritoneum (Figure 1(a)). In the MGO-treated rats, TN-C was abundant at the surface of the peritoneum where cells excessively proliferated (Figure 1(b)). In the FA-treated rats, there was slight deposition of TN-C at the surface of the peritoneum where cells were lost (Figure 1(c)).

**Figure 2:**
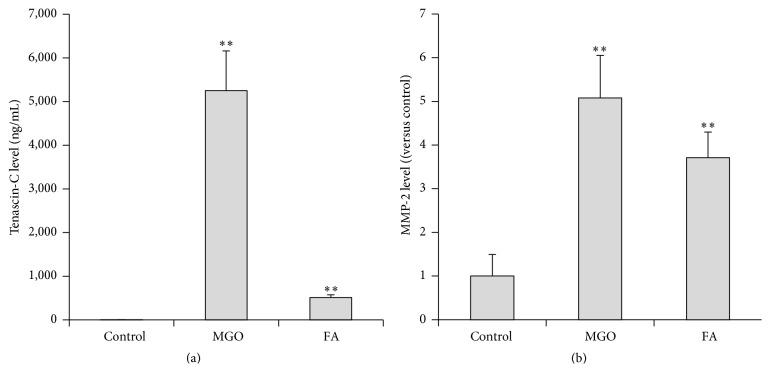
Effluent levels of TN-C and MMP-2 in the MGO- or FA-treated rats. (a) TN-C levels in the drained dialysate. (b) MMP-2 levels in the drained dialysate. Each column represents the mean ± SD of 6 rats. ^*∗∗*^ *p* < 0.01 compared with control.

The effluent TN-C levels of the MGO- or FA-treated rats increased approximately 3000 times and 300 times, respectively (Figure 2(a)). The effluent MMP-2 levels of the MGO- and FA-treated rats increased 5 and 3.7 times, respectively (Figure 2(b)).

### 3.2. Relationships between the Peritoneal Solute Transport Rate and TN-C or MMP-2 Levels in the Drained Dialysate Prepared from Patients

**Figure 3:**
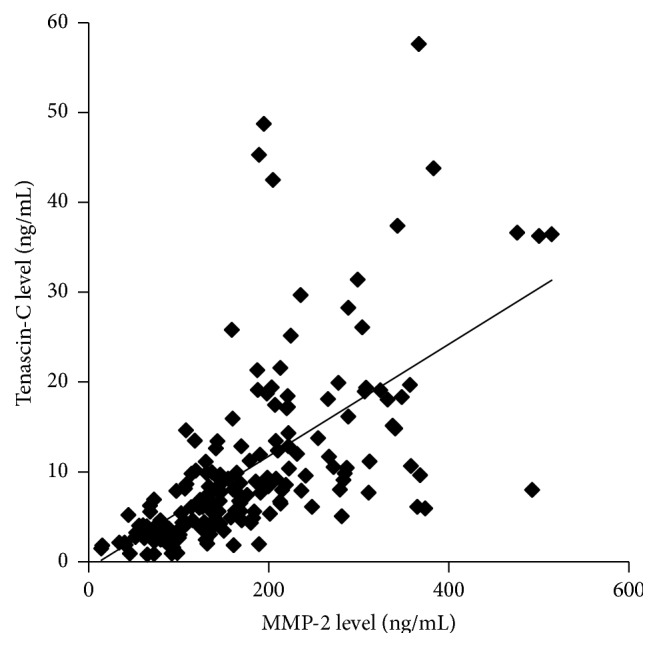
The relationship between TN-C and MMP-2 levels in the drained dialysate.

**Figure 4:**
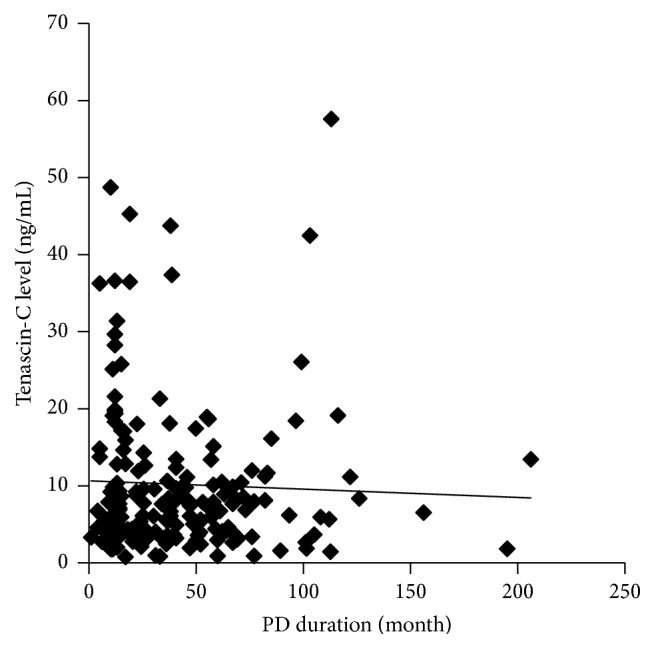
The relationship between PD duration and TN-C levels in the drained dialysate.

A total of 182 PD patients at 18 centers in Japan were analyzed. Patient characteristics are summarized in Table 1. TN-C and MMP-2 levels in the drained dialysate are shown as median (interquartile range) of 7.1 ng/mL (4.0–12.4 ng/mL) and 150 ng/mL (103–221 ng/mL), respectively. The levels of TN-C and MMP-2 in the drained dialysate highly correlated with the peritoneal solute transport rate determined by the PET (Table 2). The correlation coefficient between effluent TN-C and MMP-2 levels (*ρ* = 0.75, *p* < 0.001) was higher than that between these biomarker levels and the results of the PET (Figure 3 and Table 2). These biomarker levels significantly correlated with the numbers of peritonitis episodes, but not PD duration (Figure 4 and Table 3). The relationships between the effluent biomarker levels and the characteristics of the patients are shown in Table 3.

**Figure 5:**
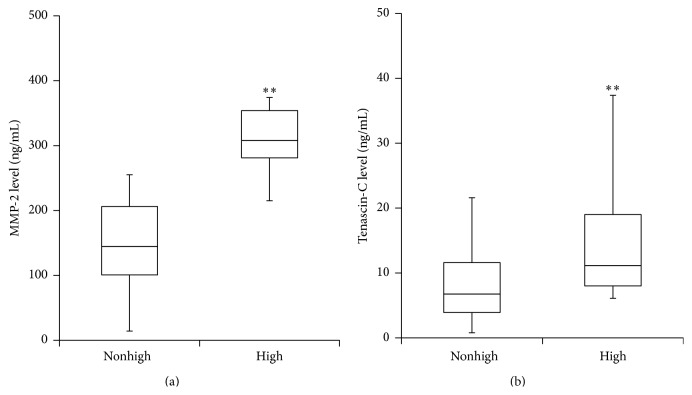
The effluent biomarker levels of patients in the high category of the PET. (a) Effluent TN-C levels. (b) Effluent MMP-2 levels. Data are shown as medians with the spread from 25th to 75th percentile (box). ^*∗∗*^ *p* < 0.01 compared with the nonhigh category group.

The proportion of high-PET-category patients in the present study was 8% of PD patients. TN-C and MMP-2 levels in the drained dialysate from high-PET-category patients were significantly higher than those from non-high-PET-category patients (Figure 5). To assess the ability of the biomarkers to diagnose the high category of the PET, ROC curves were constructed. On the ROC curves, the cut-off points of TN-C and MMP-2 for the high category of the PET were 5.7 ng/mL and 213 ng/mL, respectively (Table 4). The areas under the ROC curve (AUC) for effluent TN-C and MMP-2 levels against the high category of the PET were 0.71 and 0.91, respectively (Table 4).

Serum TN-C levels were analyzed in 20 patients (median age: 58 years, median PD duration: 15 months, 75% males, 20% with diabetes). The levels of TN-C in the serum were higher than those in the drained dialysate. The regression line was calculated based on least squares regression analysis between the measured D/S ratios of the serum proteins, such as beta 2-microglobulin, MMP-3, albumin, transferrin, and IgG, and their molecular weights. The measured D/S ratio of TN-C was also plotted in relation to the molecular weight. The slope of the regression line represented the size selectivity of the peritoneal membrane; however, the measured D/S ratio of TN-C considerably exceeded the regression line (*p* < 0.01). Regression lines for each individual patient were also calculated. Based on each regression line, the expected D/S ratios of TN-C were predicted assuming that their levels in the dialysate would be determined by transport only from the circulation. In all patients, the measured D/S ratios of TN-C considerably exceeded the expected D/S ratios calculated based on the regression line of each individual patient (data not shown).

## 4. Discussion

It is important to monitor peritoneal deterioration with structure changes and functional decline, such as an increase of the peritoneal solute transport rate. Functional decline of the peritoneum can be assessed with the PET, but there is no current method to evaluate noninvasively structure changes of the peritoneum. Peritoneal deterioration, whose mechanism is not well known, may develop through multiple factors, such as infectious peritonitis and continuous exposure to unphysiologic PD fluid with low pH, high osmolarity, high concentration of glucose, and glucose degradation products (GDPs), such as MGO, glyoxal, FA, 3-deoxyglucosone (3-DG), and 3,4-dideoxyglucosone-3-ene (3,4-DGE) [2, 3, 12]. Therefore, we explored the biomarkers for peritoneal deterioration using the GDPs-treated rats [12–14]. In the present study, the PD fluids containing 20 mM MGO or FA were injected to rat. On the other hand, the concentrations of MGO and FA are 2 to 33 *μ*M and 6 to 11 *μ*M, respectively, in conventional commercial PD fluids [22–24]. The MGO and FA concentrations administered to rats were 1,000–10,000 times higher than those found in commercial PD fluids. We previously confirmed the permitted daily exposure for GDPs concentrations administered to rats in the recommended guideline by the International Conference on Harmonisation (Guideline for Residual Solvents Q3C, 1997) [12, 13]. Moreover, conventional commercial PD fluids contain various toxic GDPs, such as glyoxal, 3-DG, and 3,4-DGE, in addition to MGO and FA. Oh et al. showed in a cell culture experiment with human peritoneal mesothelial cells that 15 *μ*M MGO changed the expression of EMT markers, E-cadherin and *α*-smooth muscle actin, and in rat animal models that 27.3 *μ*M MGO in combination with other GDPs, such as glyoxal, FA, and 3-DG, induced EMT-like change in mesothelial cells [25]. These findings suggest that the morphological changes induced in the present animal experiment can be extrapolated to clinical settings.

In the present and our previous animal studies, MGO and FA resulted in peritoneal deterioration with structural changes and functional decline [12–14]. In the MGO-treated rats, effluent TN-C levels were extremely high and, at the surface of the peritoneum where collagen was scarce, TN-C-positive mesenchymal-like cells markedly proliferated. We previously reported the possibility that these cells were transformed from mesothelial cells, such as by EMT-like change via TGF-*β*1 [13]. Liu et al. showed that miR-30b is involved in MGO-induced EMT of peritoneal mesothelial cells in rats; miR-30b directly inhibited bone morphogenetic protein-7 (BMP-7) by binding to its 3′-untranslated region, causing unavailability of BMP-7 that might be the antagonist of TGF-*β*1-induced EMT [26]. From these reports, MGO may induce EMT-like change in mesothelial cells via TGF-*β*1. Che et al. reported that MGO and 3-DG, reactive dicarbonyl metabolites in the glyoxalase system and glycation reaction, respectively, selectively induced heparin-binding epidermal growth factor-like growth factor (HB-EGF) in a dose- and time-dependent manner by increasing the intracellular peroxide levels in rat aortic smooth muscle cells (SMC) [27]. However, platelet-derived growth factor, another known growth factor of SMC, was not induced by both dicarbonyls. In addition, the signal transduction by MGO and 3-DG was not mediated by protein kinase C. Stoll et al. demonstrated that expression of HB-EGF increased keratinocyte migration and invasiveness in monolayer culture [28]. Coincident with these changes, HB-EGF significantly decreased several epithelial markers including keratins 1, 5, 10, and 14 while increasing expression of markers of cellular motility including SNAI1, ZEB1, COX-2, and MMP-1. HB-EGF induced expression of the mesenchymal protein vimentin and decreased expression of E-cadherin, as well as nuclear translocation of *β*-catenin. They also showed that HB-EGF was strongly induced in regenerating the epidermis after partial-thickness wounding of human skin [28]. Taken together, their data suggested that expression of HB-EGF in human keratinocytes triggered a migratory and invasive phenotype with many features of EMT. From these reports, MGO may induce EMT-like change in mesothelial cells via HB-EGF.

MGO is a potent promoter of the production of advanced glycation end-products (AGEs). In a previous study using rats, the administration of 20 mM MGO resulted in the accumulation of AGEs, such as imidazolone and carboxyethyl lysine, in the peritoneum [29]. AGEs and MGO induced the expression of the receptor for AGE (RAGE), and then AGE-RAGE interaction induced EMT-like change in mesothelial cells via upregulation of expression of transforming growth factor-*β* (TGF-*β*) and vascular endothelial growth factor (VEGF) [24, 30, 31]. We have previously showed that, in 20 mM MGO-treated rats, AGE was detected in mesothelial cells with EMT-like change at the surface of the peritoneum and the gene expressions of RAGE, TGF-*β*, and VEGF were enhanced in the peritoneum [12, 13]. Thus, EMT-like change in mesothelial cells was induced in response to signals from RAGE binding with AGE. These reports suggest that MGO induces EMT-like change in mesothelial cells not only directly but also indirectly via formation of AGEs.

In the FA-treated rats, peritoneal injury with cellular infiltration was induced; however, cells were lost at the surface of the peritoneum. The peritoneum was fibrous thickening with dense collagen fibers [12–14]. The pathological picture of the peritoneum of the FA-treated rats resembles that of the chlorhexidine gluconate-treated rats [13, 14]. These peritoneal changes may be generally induced by toxic agents.

Effluent TN-C levels in the FA-treated rats increased but were only approximately one-tenth of the levels of the MGO-treated rats. In contrast, effluent levels of MMP-2, a biomarker of peritoneal injury, were extremely high in both MGO- and FA-treated rats. From these results, the effluent TN-C levels reflected not only peritoneal deterioration but also especially EMT-like change in mesothelial cells in the animal study.

In the present clinical study, the measured D/S ratio of TN-C was significantly higher than the expected ratio if TN-C in the effluent was transported only from the circulation. The difference between the measured D/S ratio and the expected ratio may be attributable to the local production of TN-C in the peritoneal tissue rather than the transport of TN-C from the circulation [8–10]. In addition, in the present animal study, mesothelial cells with EMT-like change that proliferated at the surface of the peritoneum showed positive signals on immunohistochemical analysis using the anti-TN-C antibody. Most TN-C in the drained dialysate may be produced from these cells in the peritoneum and effluent TN-C levels may reflect EMT-like change in mesothelial cells. From these results, effluent TN-C could be an effective diagnostic indicator of peritoneal deterioration with structural changes, particularly of mesothelial cells with EMT-like change. To confirm this hypothesis, in a clinical study we should examine the correlation between the effluent TN-C levels and EMT-like change in mesothelial cells by cytopathological analysis using biopsy of peritoneum. However, this would have been difficult to conduct in the present study because of invasive sampling.

In the present multicenter clinical study, the peritoneal transport rate estimated by the PET closely correlated with effluent TN-C levels; however, the correlation coefficient of the TN-C levels was lower than that with the MMP-2 levels. Effluent MMP-2 levels were able to predict, with high sensitivity and specificity, the high category of the PET, which is a risk factor for EPS [5]. In our previous clinical studies, effluent MMP-2 levels were high in patients with peritoneal injury [7]. In many animal studies, effluent MMP-2 levels increased in various peritoneal injury animal models with or without EMT-like change in mesothelial cells [9, 12, 13, 25]. We suggest that MMP-2 should serve as a superior indicator of general peritoneal deterioration.

In the present clinical study, the correlation coefficient between the effluent levels of TN-C and MMP-2 was high. MMP-2 and the degradation of TN-C are associated with tumor recurrence in early-stage non-small cell lung cancer [32]. TN-C deposition into the extracellular matrix requires the participation of MMP-2 and the resulting deposited TN-C promotes pancreatic cancer progression [33]. In peritoneal deterioration, TN-C may promote tissue injury in concert with MMP-2.

Effluent TN-C levels correlated with the peritoneal solute transport rate, but not PD duration in the present cross-sectional study. Confirmation that the change in effluent TN-C levels reflects increased peritoneal solute transport or EMT-like change in mesothelial cells is necessary to reach the conclusion that TN-C is a useful biomarker for early-stage peritoneal deterioration. In our longitudinal analysis, peritoneal solute transport did not develop significantly faster during the period of the present study. Further longitudinal analysis is needed.

## 5. Conclusion

Effluent TN-C levels correlated with the peritoneal solute transport rate. Most TN-C in the drained dialysate is thought to be produced by mesothelial cell-derived mesenchymal cells at the surface of the peritoneum. Therefore, effluent TN-C has a possibility of biomarker for EMT-like change in mesothelial cells in PD. Because EMT-like change in mesothelial cells is thought to be a trigger of peritoneal injury, TN-C may be useful as an indicator for early-stage peritoneal deterioration, whereas effluent MMP-2 levels reflect general peritoneal deterioration. To test this hypothesis, further longitudinal studies are necessary to examine the change in effluent TN-C levels during the progression of peritoneal deterioration with increased peritoneal solute transport.

## Figures and Tables

**Table 1:** Characteristics of patients.

Sex (male/female)	104/78 (57% male)
Etiology (nondiabetes/diabetes)	147/35 (19% diabetes)
Age (years)	58 years (47–66 years)
PD duration (months)	33 months (13–59 months)
Peritonitis episode (times)	0 times (0-1 time)
D/P Cr	0.65 (0.59–0.71)
D/D0 glucose	0.40 (0.34–0.44)
Effluent tenascin-C level	7.1 ng/mL (4.0–12.4 ng/mL)
Effluent matrix metalloproteinase-2 level	150 ng/mL (103–221 ng/mL)

Data except sex and etiology of renal failure are expressed as medians with interquartile ranges.

**Table 2:** Spearman's correlation coefficients between effluent biomarker levels and results of the PET.

	Tenascin-C	Matrix metalloproteinase-2
D/P Cr	*ρ* = 0.57	*ρ* = 0.73
	*p* < 0.001	*p* < 0.001

D/D0 glucose	*ρ* = −0.43	*ρ* = −0.63
	*p* < 0.001	*p* < 0.001

*ρ* values indicate relationships between effluent biomarker levels and results of the PET by Spearman's correlation coefficient.

**Table 3:** The correlation coefficients between patient characteristics and effluent biomarker levels.

	Biomarker levels in the peritoneal effluents	
	Tenascin-C	Matrix metalloproteinase-2
Sex (male/female)	*p* = 0.62	*p* = 0.50
Etiology (nondiabetes/diabetes)	*p* = 0.12	*p* = 0.30
Age (years)	*ρ* = 0.048, *p* = 0.51	*ρ* = 0.026, *p* = 0.73
PD duration (months)	*ρ* = −0.070, *p* = 0.34	*ρ* = 0.017, *p* = 0.82
Peritonitis episode (times)	*ρ* = 0.15, *p* < 0.05	*ρ* = 0.21, *p* < 0.01

*ρ* values: Spearman's correlation coefficient.

**Table 4:** Test performance of effluent biomarker cut-off levels to diagnose the high category of the PET.

Biomarker	Cut-off level	Sensitivity	Specificity	False negative	AUC
Tenascin-C	5.7 ng/mL	100%	42%	0%	0.71
	6.0 ng/mL	92%	43%	1.3%	
	6.5 ng/mL	85%	48%	3.6%	

Matrix metalloproteinase-2	213 ng/mL	93%	79%	0.8%	0.91
	250 ng/mL	86%	87%	1.4%	
	270 ng/mL	79%	88%	2.0%	

AUC: area under curve.
